# Dietary vitamin A modifies the gut microbiota and intestinal tissue transcriptome, impacting intestinal permeability and the release of inflammatory factors, thereby influencing Aβ pathology

**DOI:** 10.3389/fnut.2024.1367086

**Published:** 2024-03-27

**Authors:** Zhong-Li Wang, Shao-Jie Pang, Kai-Wen Zhang, Peng-Yu Li, Peng-Gao Li, Chun Yang

**Affiliations:** ^1^Department of Rehabilitation Medicine, The Second Affiliated Hospital of Jiaxing University, The Second Hospital of Jiaxing, Zhejiang, China; ^2^Heilongjiang Feihe Dairy Co., Ltd. Feihe Research Institute, Beijing, China; ^3^School of Public Health, Capital Medical University, Beijing, China; ^4^Institute of Food Science and Technology, Chinese Academy of Agricultural Sciences, Beijing, China

**Keywords:** Alzheimer’s disease, vitamin A, gut microbiota, intestinal permeability, inflammatory factors, Aβ pathology

## Abstract

**Background:**

Alzheimer’s disease (AD) is an age-related neurodegenerative disorder with no effective interventions for curing or modifying its progression. However, emerging research suggests that vitamin A in the diet may play a role in both the prevention and treatment of AD, although the exact mechanisms are not fully understood.

**Objectives:**

This study aims to investigate the dietary vitamin A modifies the gut microbiota and intestinal tissue transcriptome, impacting intestinal permeability and the release of inflammatory factors, thereby influencing Aβ pathology shedding light on its potential as a dietary intervention for AD prevention and treatment.

**Methods:**

The APP/PS1-AD mouse model was employed and divided into three dietary groups: vitamin A-deficient (VAD), normal vitamin A (VAN), and vitamin A-supplemented (VAS) for a 12-week study. Neurobehavioral functions were assessed using the Morris Water Maze Test (MWM). Enzyme-linked immunosorbent assay (ELISA) was used to quantify levels of Diamine Oxidase (DAO), D-lactate, IL-6, IL-1β, and TNF-a cytokines. Serum vitamin A levels were analyzed via LC-MS/MS analysis. Immunohistochemical analysis and morphometry were performed to evaluate the deposition of Aβ in brain tissue. The gut microbiota of APP/PS1 mice was analyzed using 16S rRNA sequencing analysis. Additionally, transcriptomic analysis was conducted on intestinal tissue from APP/PS1 mice.

**Results:**

No significant changes in food intake and body weight were observed among the groups. However, the VAD and VAS groups showed reduced food intake compared to the VAN group at various time points. In terms of cognitive function, the VAN group performed better in the Morris Water Maze Test, indicating superior learning and memory abilities. The VAD and VAS groups exhibited impaired performance, with the VAS group performing relatively better than the VAD group. Serum vitamin A concentrations differed significantly among the groups, with the VAS group having the highest concentration. Aβ levels were significantly higher in the VAD group compared to both the VAN and VAS groups. Microbial analysis revealed that the VAS and VAN groups had higher microbial diversity than the VAD group, with specific taxa characterizing each group. The VAN group was characterized by taxa such as Actinohacteriota and Desulfovibrionaceae, while the VAD group was characterized by Parabacteroides and Tannerellaceae. The VAS group showed similarities with both VAN and VAD groups, with taxa like Desulfobacterota and Desulfovibrionaceae being present. The VAD vs. VAS, VAD vs. VAN, and VAS vs. VAN comparisons identified 571, 313, and 243 differentially expressed genes, respectively, which associated with cellular and metabolic processes, and pathway analysis revealed enrichment in pathways related to chemical carcinogenesis, drug metabolism, glutathione metabolism, and immune-related processes. The VAD group exhibited higher levels of D-lactate, diamine oxidase, and inflammatory cytokines (TNF-a, IL-1β, IL-6) compared to the VAN and VAS groups.

**Conclusion:**

Dietary vitamin A supplementation modulates the gut microbiota, intestinal permeability, inflammatory factors, and Aβ protein formation, offering insights into the pathogenesis of AD and potential therapeutic avenues for further exploration. This research highlights the intricate interplay between diet, gut microbiota, and neurodegenerative processes, emphasizing the importance of dietary interventions in managing AD-related pathologies.

## Introduction

Alzheimer’s disease (AD) is widely recognized to be an age-related neurodegenerative disease, deposits of amyloid-β (Aβ) and hyperphosphorylated tau protein are its main characteristics (1). Recent data from the 2021 Alzheimer’s World Report indicated that more than 55 million people worldwide have dementia (2). About 60% of those dementia patients lived in low- and middle-income countries. Meanwhile, China, India, and their South Asian and Western Pacific neighbors have the fastest growing rate of elderly populations. Considering the anticipated patterns of population aging, it is expected that the prevalence of AD and the corresponding financial burden will increase. Currently, there is a lack of efficacious interventions capable of curing AD or modifying the pathological progression in the brain. Consequently, the effective regulation and management of risk factors associated with AD have assumed paramount significance. Diet has been implicated as a crucial factor in both the prevention and progression of AD (3). Vitamin A, a fat-soluble micronutrient and its metabolite retinoic acid (RA), has emerged as a potential preventive and therapeutic strategy for AD, neuroplasticity, essential for learning and adaptation, relies on adequate vitamin A intake and RA signaling. Furthermore, non-genomic actions of vitamin A and RA within the brain play a crucial role in AD prevention and treatment. Modulating these pathways may enhance brain function and offer novel therapeutic avenues (4). In spite of this, it is still unclear how vitamin A prevents and treats AD. This study aims to investigate the dietary vitamin A modifies the gut microbiota and intestinal tissue transcriptome, impacting intestinal permeability and the release of inflammatory factors, thereby influencing Aβ pathology shedding light on its potential as a dietary intervention for AD prevention and treatment.

## Methods

The animal experiments were approved by the Animal Ethics Committee of Capital Medical University (Beijing, China) (No. AEEI-2022-236). Thirty male APP/PS1 mice (4 weeks old) obtained from Hua Fukang Biological Technologies (Beijing, China) were acclimatized to the new environment for 1 week. The APP/PS1 mice were randomly divided into three groups according to body weight, namely VAD, VAN, and VAS groups. The animals were fed with AIN93G diet supplemented with different amounts of vitamin A: 0 IU/g (VAD), 4 IU/g (VAN), or 15 IU/g (VAS).

All feed was provided by Beijing Kefao Xieli Feed Co., Ltd. (Beijing, China). During the experiment, they were exposed to 12 h of light and 12 h of darkness, maintained at a constant temperature of 20°C, and supplied with food and water on a constant basis. Animals were observed twice a week, and food intake and body weight were recorded weekly. After a 12-week period, the mice were euthanized by cervical dislocation under anesthesia following a 12-h fasting protocol prior to decapitation.

### Collection and preservation of samples

Mice fecal samples were obtained during the 8:00 to 10:00 AM time frame at the conclusion of the 12th week and placed in pre-labeled sterile collection tubes. These samples were subsequently stored at a temperature of −80 degrees Celsius for further analysis. Serum samples were obtained from anesthetized mice through intraperitoneal injection of sodium pentobarbital, followed by blood extraction from their eyeballs after the removal of the eyeballs. Subsequently, the mice were dissected, and tissue from the brain and gut was collected. Brain tissues were extracted from animals transcardially perfused with phosphate-buffered saline (PBS), followed by fixation with 4% paraformaldehyde for 30 min. The small intestine (a 10-cm segment from the upper part of the small intestine) was isolated and flushed with PBS. The tissue was promptly frozen in liquid nitrogen and then transferred to a temperature of −80°C for preservation after being weighed, the tissue was promptly frozen in liquid nitrogen and subsequently transferred to a temperature of −80°C for the purpose of preservation. The blood samples were maintained within a refrigerator set at a temperature of 4°C for a duration of 30 min, after which they were subjected to centrifugation at a speed of 3,000 rpm for a period of 15 min. The resulting serum was then carefully transferred to a fresh centrifuge tube and stored at a temperature of −80°C.

### Enzyme-linked immunosorbent assay

Serum levels of markers of intestinal permeability, such as Diamine Oxidase (DAO) and D-lactate, were measured using a mouse-specific ELISA kit, while serum cytokines including IL-6, IL-1β, and TNF-α were assessed with ELISA kits from MEIMIAN, China, according to the manufacturer’s instructions.

### Morris water maze test

A six-day water maze experiment was conducted, wherein mice underwent four days of training followed by a 60-s search for a hidden platform from four different quadrants. In order to assess the mice’s memory of the platform’s location, they were briefly placed on the platform for 5 s after each test if they were unable to locate it. Subsequently, a 60-s detection experiment was carried out after the mice had completed five days of training, during which the time taken to find the hidden platform (escape latency) was measured. On the concluding day of the experiment, two trials were performed: the first entailed conducting the no platform test, wherein mice were assessed for their ability to locate the platform position within a span of 60 s, without the presence of a platform. The second experiment involved measuring the duration it took for the mice to locate the platform, employing the platform test.

### Immunohistochemical analysis and morphometry

In this experiment, mouse brains were fixed in 4% paraformaldehyde in PBS for 24 h, dehydrated, and embedded in paraffin wax. The serial sections were cut at a thickness of 4 mm. Sections were microwaved (700 W) in citrate buffer pH 6.0 for 18 min, then digested with 100 g/mL proteinase K (Worthington) in TBS for 6 min at 37°C. A biotinylated anti-mouse IgG antibody (Vector Laboratories) was incubated with the sections after blocking with 10% calf serum in TBS. Then, avidin-biotin complex (ABC elite, Vector Laboratories) is used as chromogen for visualization. Counterstained with hematoxylin, the sections were lightly stained. The quantification of the percentage of Aβ-positive areas relative to the total area (referred to as Aβ burden), was conducted using the Image J software (NIH) (5). Aβ-positive areas exhibiting brown coloration above a predetermined threshold were binarized against the negative areas, and the percentage of positive area coverage was calculated.

### The gut microbiota in the feces of APP/PS1 mice was analyzed using 16S rRNA sequencing analysis

Library construction and sequencing: after extracting total DNA of samples, primers were designed according to the conserved region. Sequencing adapters were added to the end of primers. The target sequences were amplified by PCR and its products were purified, quantified and homogenized to get a sequencing library. Then library QC was performed for constructing libraries. Qualified libraries were sequenced on Illumina Novaseq 6000. The original image data files obtained by high-throughput sequencing (such as Illumina Novaseq and other sequencing platforms) were converted into Sequenced Reads by Base Calling analysis. The results were stored in FASTQ (referred to as fq) format file, which contains sequence information of reads and their corresponding sequencing quality information.

Raw data processing includes following 2 steps: (1) Raw reads filtration: Raw reads were firstly filtered by Trimmomatic v 0.33 then the primer sequences were identified and removed by cutadapt 1.9.1, which finally generated high-quality reads without primer sequences; (2) DADA 2 de-noise: De-noise were processed by dada 2 (6) in R library in order to de-noise and remove chimeric sequences, generating non-chimeric reads. Bioinformatic analysis includes: Alpha diversity was calculated by the diversity indices (Shannon). Principal Component Analysis (7) for beta diversity analysis and LEfSe (8). Line Discriminant Analysis (LDA) Effect Size is an algorithm for biomarker discovery between groups, which combines non-parametric Kruskal-Wallis and Wilcoxon sum-rank test with effective size estimated linear discriminant analysis.

### Transcriptomic analysis of intestinal tissue in APP/PS1 mice

We employed the NanoDrop 2000 spectrophotometer for assessing the purity and concentration of RNA, while the Agilent 2,100/Lab Chip GX system was used for accurate detection of RNA integrity. The library construction process involved the enrichment of eukaryotic mRNA using magnetic beads with Oligo (dT). The mRNA was then randomly fragmented using a Fragmentation Buffer. First-strand cDNA synthesis, as well as second-strand synthesis, was performed using the mRNA as a template, followed by cDNA purification. The purified double-stranded cDNA underwent end repair, a-tailing, and adaptor ligation. Subsequently, fragment size selection was carried out using AMPure XP beads. Finally, cDNA libraries were enriched through PCR. After the completion of library construction, initial quantification was performed using the Qubit 3.0 fluorometer, with a required concentration of above 1 ng/μl. Subsequently, the Qsep400 high-throughput analysis system was utilized to evaluate the insert sizes of the libraries. Upon confirming the expected insert sizes, the Q-PCR method was employed for precise quantification of the library’s effective concentration (library effective concentration > 2 nM) to ensure library quality. For sequencing, the Illumina NovaSeq6000 platform was used in PE150 mode. Following sequencing, the raw data was filtered to obtain clean data, which was then aligned to a specified reference genome to generate mapped data. Raw data analysis was performed using BMKCloud.^1^ The differentially expressed genes (DEGs) between the comparison groups were obtained by standardizing processing and screening conditions. Criteria for differentially expressed genes was set as Fold Change (FC) ≥ 1.5 and *p* value < 0.01. Fold change (FC) refers to the ratio of gene expression in two samples. False Discovery Rate (FDR) refers to adjusted *p*-value, which is used to measure significancy of difference. The KEGG database was used for functional annotation, classification statistics, and metabolic pathway analysis of DEGs in the comparison group.

### Serum vitamin A assay

The detection method for serum vitamin A in APP/PS1 mice following steps. Transfer 50 μL of the sample to a 1.5 mL centrifuge tube. Add 200 μL of precipitating agent containing an internal standard (500 ng/mL Retinol-d4, prepared in acetonitrile) and vortex for 3 min. Centrifuge at 15,000 rpm for 10 min, and transfer 150 μL of the supernatant to an injection vial. Take 10 μL of the supernatant for LC–MS/MS analysis was performed using AB SCIEX Quad™ 4500MD. The separation and analysis conditions were as follows: SHIMADZU Shim-pack GIST-HP C8(50*2.1 mm, 3 μm), mobile phase: 0.1% formic acid solution (A), 0.1% formic acid methanol (B), gradient elution program: 0–1.5 min, 80% B, 1.5–2.0 min, 95% B, 2.0–2.5 min, 100% B, 2.5–3.0 min, 80% B. 0–0.3 min and 2.5–3.0 min flow rate is 0.6 mL/min, while the flow rate is 0.8 mL/min for the remaining time, column temperature: constant temperature 40°C, sample room temperature: constant temperature 10°C.

### Statistical methods

Statistical analysis was conducted using IBM SPSS 26.0 software (SPSS Inc.). The data was presented as means and standard deviation (±SD), and statistical analyses were performed using one-way analysis of variance (one-way ANOVA) and repeated measures ANOVA with Bonferroni’s *post-hoc* test to compare multiple repeated measurements between groups. Statistical significance was defined as a *p* value < 0.05, <0.01, or 0.001.

## Results

### The impact of vitamin A on dietary intake and weight changes in APP/PS1 mice

**Figure 1:**
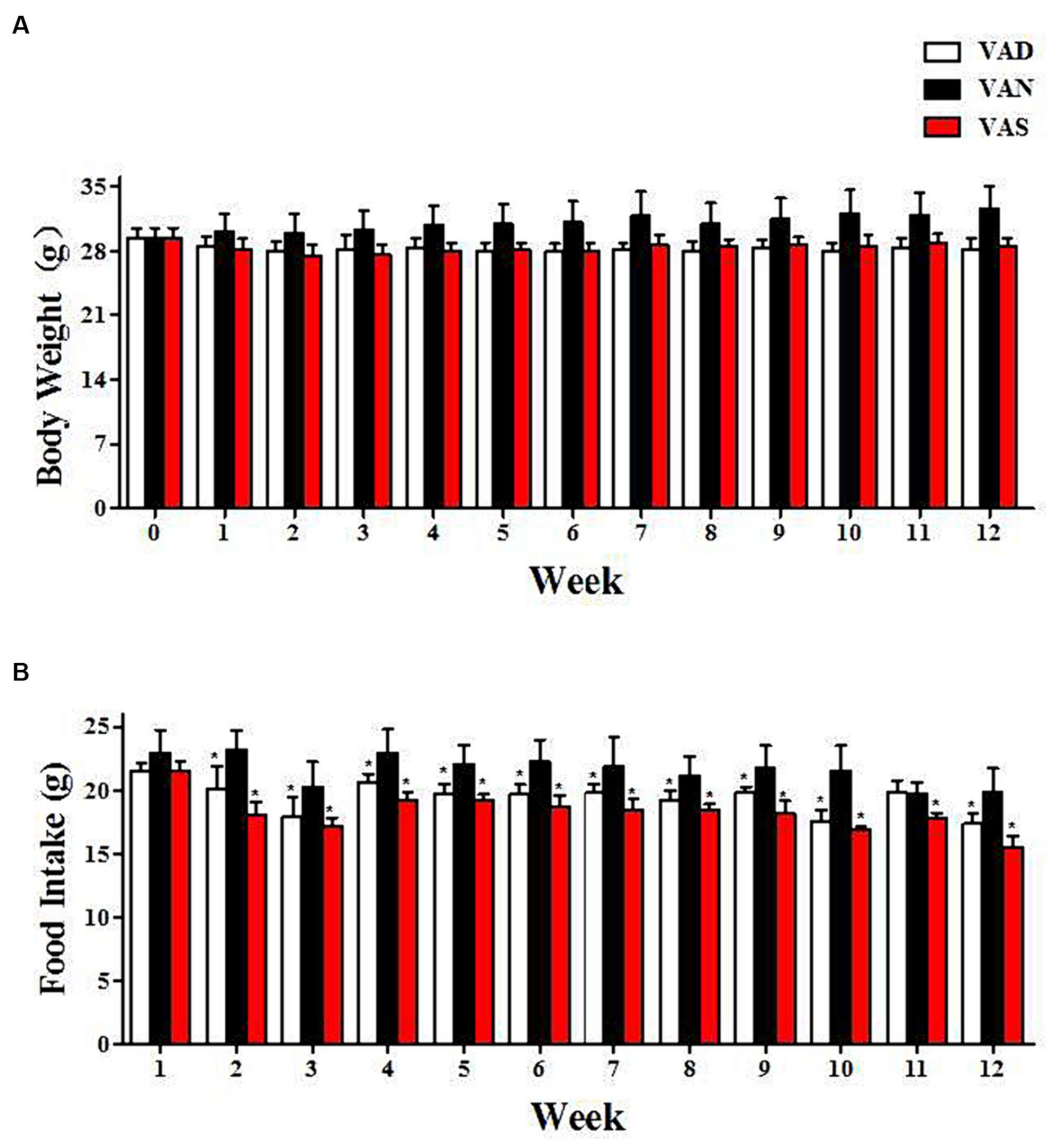
The impact of vitamin A on dietary intake and body weight changes in APP/PS1 mice. **(A)** The changes in body weight of APP/PS1 mice among different groups. **(B)** The changes in dietary vitamin A intake among different groups of APP/PS1 mice. *Indicates compared with the VAN group, *p* < 0.05 indicates statistical significance.

Throughout the entirety of the experimental period, Figure 1 demonstrates that there were no noteworthy alterations in body weight and dietary consumption among APP/PS1 mice subjected to various dietary interventions. Nevertheless, at distinct time intervals, both the VAD and VAS groups exhibited reduced body weight (Figure 1A) and food intake (Figure 1B) in comparison to the VAN group. Despite the lack of statistical significance in body weight differences, the VAD and VAS groups exhibited a significant decrease in food intake compared to the VAN group at different time intervals.

### The impact of vitamin A on serum vitamin A in APP/PS1 mice

LC–MS/MS is utilized to measure the serum concentration of vitamin A in APP/PS1 mice. The VAD group had an average concentration of 382.61 ± 79.76 ng/mL, the VAN group had 548.32 ± 40.06 ng/mL, and the VAS group had 640.85 ± 32.44 ng/mL. The one-way ANOVA analysis revealed a significant difference between the groups, with an *F*-value of 34.16 and a *p*-value of 0.0000026. Dietary vitamin A intervention affects the levels of vitamin A on serum vitamin A in APP/PS1 mice.

### The effects of vitamin A on cognition of APP/PS1 mice

**Figure 2:**
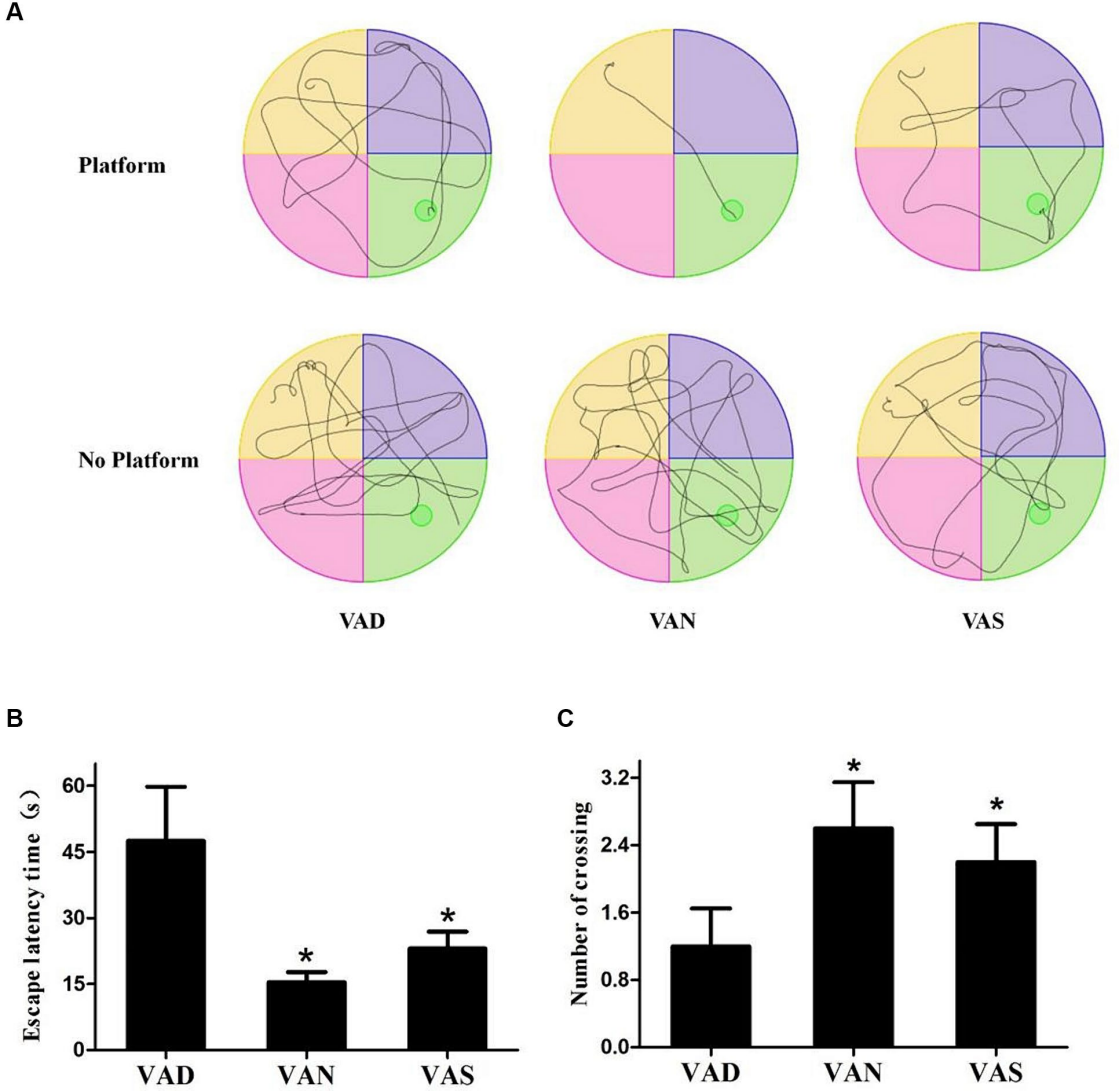
The effects of vitamin A on cognition of APP/PS1 mice. **(A)** The mean latency time taken by APP/PS1 mice of different groups to find the platform (escape latency) in the Morris water maze task. **(B)** The mean number of escape latency time among APP/PS1 mice from different groups in the Morriswater maze task. (C) The mean number of platform crossings among APP/PS1 mice from different groups in the Morris water maze task when there is no platform. All of the data were analyzed using a one-way ANOVA and they are expressed as means ± SD. *Indicates compared with the VAD group, *p* < 0.05 indicates statistical significance.

The MWM test is an effective method for detecting learning and memory abilities in mice suffering from neurodegenerative disorders, based on the MWM test, the swimming trace was determined, as well as the escape latency time on the platform location and the number of times crossing of the platform was found in the absence of a platform. Figure 2A shows the trajectories of the swimmer. Mice in a VAN group can almost directly find the platform for resting, or move to the area where there is no platform to locate the platform that has been removed. Regardless of whether there is a platform or not, the mice in the other groups have no purpose to swim around irregularly, indicating that the mice have more learning and memory disorders. Additionally, the mice in the VAN group were able to locate the platform in a more amount of time compared to the other groups, and the time taken by the other groups to find the platform was significantly (*p* < 0.01) higher than that of the VAN group, specifically, the VAS group had a shorter time to find the platform compared to the VAD group, however, there were no statistically significant differences observed between the VAN and VAS group (Figure 2B). Furthermore, in the absence of the platform, the mice in the VAN group repeatedly crossed the platform area. The number of mice in the other groups crossing for the platform was lower than that of the VAN group. Additionally, when compared to the VAD group, the VAN and VAS group showed a significant (*p* < 0.01) increase in the number of mice crossing for the platform. However, no statistically significant differences were observed between the VAN and VAS group (Figure 2C). These results suggest that vitamin A could influence the learning and memory ability of APP/PS1 mice.

### Effects of vitamin A on the Aβ pathology in the brain of APP/PS1 mice

**Figure 3:**
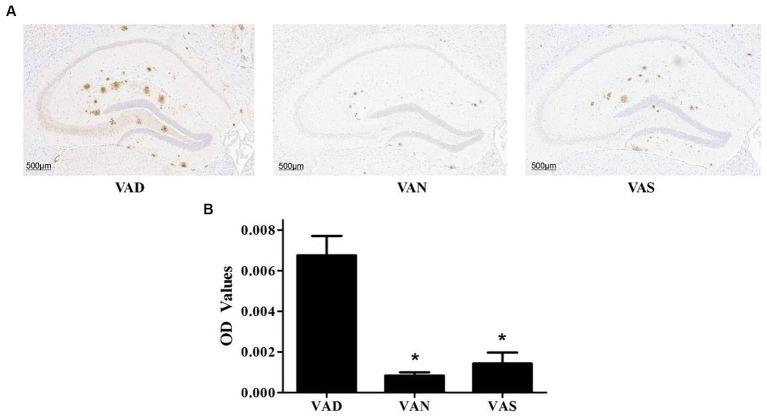
Effects of vitamin A on the Aβ pathology in the brain of APP/PS1 mice. **(A)** Representative images of immunohistochemical staining of the brains of APP/PS1 mice using an anti-Aβ (82E1) antibody in different groups. **(B)** Compare the quantification results of Aβ immunoreactivity in the hippocampus of the brains of APP/PS1 mice among different groups. All of the data were analyzed using a one-way ANOVA and they are expressed as means ± SD. *Indicates compared with the VAD group, *p* < 0.05 indicates statistical significance.

There has been study that have investigated the effects of vitamin A on the Aβ pathology of APP/PS1 mice (Figure 3). The VAD group exhibits a significantly greater deposition of Aβ compared to both the VAN and VAS groups (Figure 3A), with statistical significance. While there is no statistically significant difference in Aβ deposition between the VAN and VAS groups, it is noteworthy that the VAS group demonstrates a higher level of Aβ deposition in comparison to the VAN group (Figure 3B) The results of the study have shown that vitamin A supplementation can affect the levels of Aβ in the brain of APP/PS1 mice.

### Effects of vitamin A on the intestinal microflora of APP/PS1 mice

**Figure 4:**
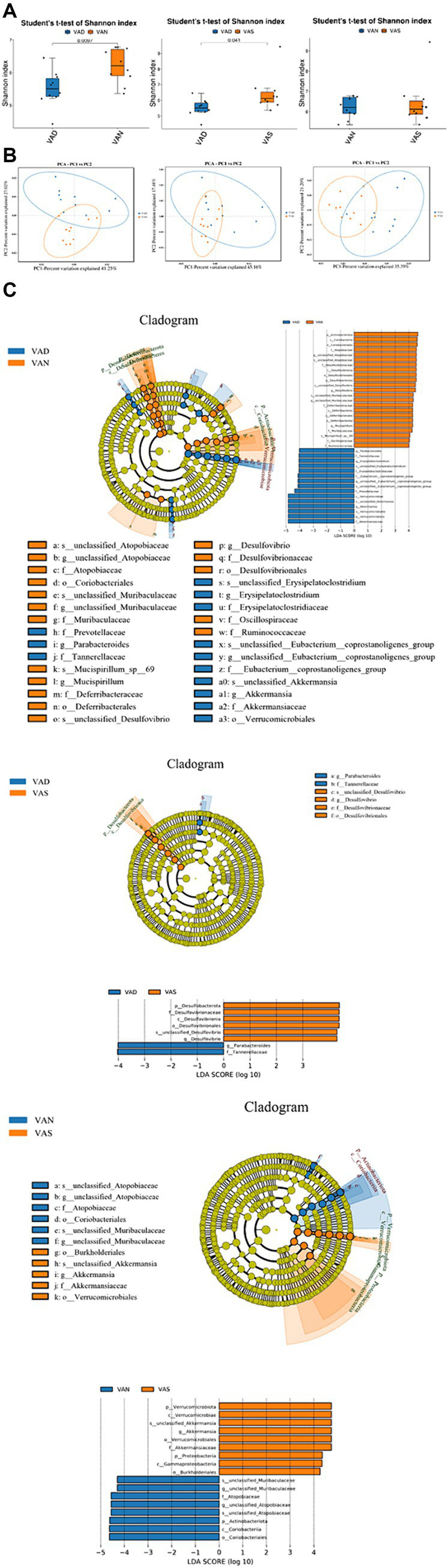
Effects of vitamin A on the intestinal microflora of APP/PS1 mice. **(A)** Comparison of the Shannon index of gut microbiota in different groups of APP/PS1 mice. Significant differences were determined using the Student’s *t*-test as the default method for testing significance (*p* < 0.05). **(B)** Comparison of Principal Component Analysis of gut microbiota in different groups of APP/PS1 mice. Each dot represents a sample. The samples were colored based on grouping information if applicable. The confidence ellipse defines the region that contains 95% of all samples that can be drawn from underlying Gaussian distribution. X-axis: First principal component. The value in percentage indicates contribution of PC1 in variability. Y-axis: Second principal component. The value in percentage indicates contribution of PC2 in variability. **(C)** LEfSe among different groups of APP/PS1 mice in gut microbiota. Cladogram diagram: circles from center to outward layers represent taxonomic level from phylum to species. The node on circles represents a term on corresponding taxonomic level. The size of the dots indicates relative abundance. Coloring: Species with no significant difference are colored in yellow. Otherwise, the nodes were colored according to the group with the highest relative abundance, which helps visualize the relevance of different biological aspects. LDA score distribution histogram: Y-axis: Features that shown significant difference between groups; X-axis: Log10 of LDA score. The features were sorted according to LDA score. A longer bar indicates a more significant difference. The bars were colored according to the group with highest abundance of corresponding feature.

The results of Shannon indices indicated that compared to the VAD group, the VAS and VAN group have higher Shannon indices (*p* < 0.05), but the similar between VAN and VAS group. VAS and VAN group have higher diversity than VAD group (Figure 4A). The results of PCA show that there is a small overlap between the VAN group and the VAD and VAS groups, indicating a potentially distinct composition compared to the VAD and VAS groups. On the other hand, the majority of the VAD and VAS groups overlap, suggesting that they share more similarities in terms of their composition (Figure 4B). In LEfSe analysis, the computational approach to comparing biomarker classes contributes to the understanding of microbial communities (Figure 4C).

In the VAN and VAD groups, the VAN group is characterized by taxa such as p_Actinohacteriota, c_Coriabacteria, o_Coriobacteriales, f_Atopobiaceae, g_unclassified_Atopobiaceae, s_unclassified_Atopobiaceae, f_Desulfovibnonaceae, c_Desulfovibrionia, o_Desulfovibrionales, p_Desulfobacterota, s_unclassified_Desulfovibrio, g_DesLulfovibrio, s_unclassified_Muribaculaceae, g_unclassified_Muribaculaceae, f_Deferribacteraceae, c_Deferribacteres, o_Deferribacterales, p_Deferribacterota, g_Muospirillum, f_Muribdculaceae, s_Mucispirillum_sp_69, f_Oscillospiraceae, and f_Ruminococcaceae. On the other hand, the VAD group is characterized by taxa such as g_Parabacteroides, f_Tannehefellaceae, g_Erysigelatoclosttidium, s_unclassified_Erysipelatoclostridium, f_Erysipelatoclostridiaceae, f_Eubacterium_coprostanoligenes_group, g_unclassified_Eubacterium_coprostanoligenes_group, s_unclassified_Eubacterium_coprostanoligenes_group, f_Prevotellaceae, c_Verrucomicrobiae, s_unclassified_Akkermansia, g_Akkermansia, o_Verrucomicrobiales, p_Verrutomicrobidta, and f_Akkerrnansiaceae.

In the VAS and VAD groups, the VAS group is characterized by taxa such as p_Desulfobacterota, f_Desulfovibrionaceae, c_Desulfovibrionia, o_Desulfovibrionales, s_unclassified_Desulfovibrio, and g_Desulfovibrio. On the other hand, the VAD group is characterized by taxa including g_Parabacteroides and f_Tannerellaceae. In the VAN and VAS groups, the VAN group is characterized by taxa including p_Verrucomicrobiota, c_Verrucomicrobiae, s_unclassified_Akkermansia, g_Akkermansia, o_errucomicrobiales, f_Akkermansiaceae, p_Proteobacteria, c_Gammaproteobacteria, and o_Burkholderiales. Conversely, the VAS group is characterized by taxa such as s_unclassified_Muribaculaceae, g_unclassified_Muribaculaceae, f_Atopobiaceae, g_unclassified_Atdpobiaceae, s_unclassified_Atopobiaceae, p_Actinobacteriota, c_Coriobacteriia, and o_Coriobacteriales.

### Effects of vitamin A on the transcriptomic of intestinal tissue in APP/PS1 mice

**Figure 5:**
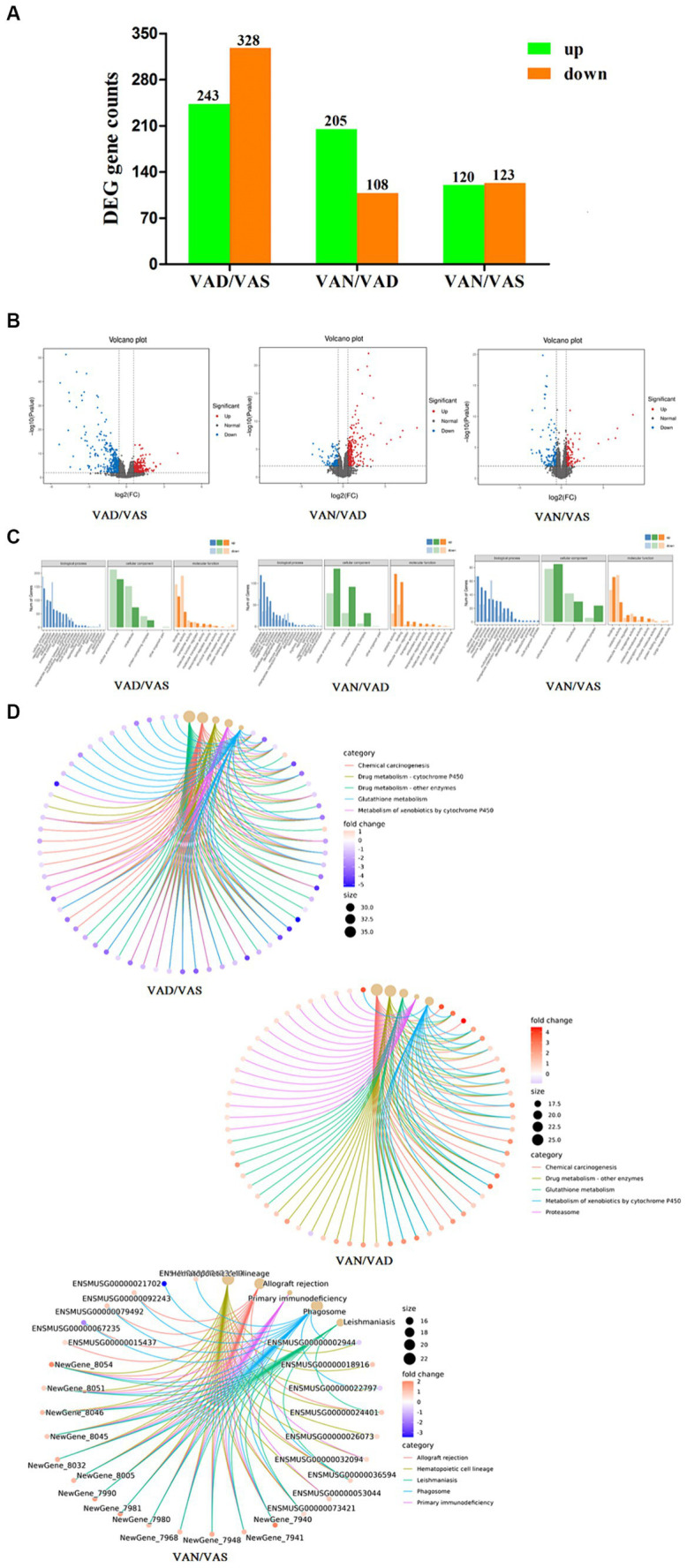
Effects of vitamin A on the transcriptomic of intestinal tissue in APP/PS1 mice. **(A)** Histogram of the number of differentially expressed genes in different groups of APP/PS1 mice. Criteria for differentially expressed genes was set as Fold Change (FC) ≥ 1.5 and *p* value < 0.01. Fold change (FC) refers to the ratio of gene expression in two samples. False Discovery Rate (FDR) refers to adjusted *p*-value, which is used to measure significancy of difference. **(B)** Volcano plot of the number of differentially expressed genes in different groups of APP/PS1 mice. Each dot represents a gene. X-axis: log2Fold change of expression; Y-axis: −log10(FDR) or −log10(*p*-value). Dots farther to y = 0 represent genes with large difference in expression between two samples. Dots farther to x = 0 represents genes of which the difference is more reliable. Green dots are down-regulated genes, while red dots are up-regulated ones and black dots are genes without significant difference. **(C)** GO classification of differentially expressed genes in different groups of APP/PS1 mice. X-axis: Go terms and classifications; Y-axis: Number of DEGs (genes) annotated to the term (right). **(D)** Cnetplot of KEGG pathways in different groups of APP/PS1 mice. The color of the edges represents different pathways, while the color of gene nodes indicates fold-change values. Larger pathway nodes indicate a greater number of genes enriched in that pathway. This figure displays the top 5 most enriched (*q*-value) pathways and their associated genes, providing a visual representation of the relationship between genes and pathways.

In the intestinal tissue of APP/PS1 mice, we used RNA sequencing technology to perform comparative analysis of vitamin A in different groups. As shown in the Figure 5A and volcano plot Figure 5B, when comparing the VAD group to the VAS group, we observed upregulation of 243 genes and downregulation of 328 genes, resulting in a total of 571 differentially expressed genes. Similarly, in the comparison between the VAD group and the VAN group, we observed upregulation of 205 genes and downregulation of 108 genes, resulting in a total of 313 differentially expressed genes. In contrast, when comparing the VAS group to the VAN group, we observed upregulation of 120 genes and downregulation of 123 genes, resulting in a total of 243 differentially expressed genes. These findings highlight the distinct gene expression profiles associated with different vitamin A interventions in the intestinal tissue of APP/PS1 mice.

As shown in the Figure 5C, GO classification of DEGs between group was shown in the figure, when comparing between the VAD group and the VAS group, it revealed that GO classification of these genes was involved in cellular processes, metabolic processes, cellular anatomical entities, intracellular activities, binding, and catalytic activities. Similarly, when comparing the VAD group to the VAN group, the GO classification of DEGs showed enrichment in cellular processes, metabolic processes, cellular anatomical entities, intracellular activities, catalytic activities, and binding. Furthermore, the comparison between the VAS group and the VAN group revealed GO classification terms related to cellular processes, system processes, cellular anatomical entities, intracellular activities, binding, and catalytic activities. These GO classification results provide insights into the functional characteristics and molecular activities associated with the differentially expressed genes in each comparison group. KEGG analysis and pathway enrichment analysis were conducted in the KEGG pathway database, as shown in the cnetplot of KEGG pathways Figure 5D results revealed the enriched pathways for the DEGs, when comparing the VAD group to the VAS group. These pathways include Chemical carcinogenesis, Drug metabolism - cytochrome P450, Drug metabolism - other enzymes, Glutathione metabolism, and Metabolism of xenobiotics by cytochrome P450. Similarly, when comparing the VAD group to the VAN group, the DEGs enriched pathways include Chemical carcinogenesis, Drug metabolism - other enzymes, Glutathione metabolism, Metabolism of xenobiotics by cytochrome P450, and Proteasome. Furthermore, the comparison between the VAD group and the VAN group revealed the DEGs enriched pathways as Allograft rejection, Hematopoietic cell lineage, Leishmaniasis, Phagosome, and Primary immunodeficiency. These findings provide insights into the potential biological pathways and processes associated with the differentially expressed genes in each comparison group.

### Effects of vitamin A on intestinal mucosal permeability and inflammation in APP/PS1 mice

**Figure 6:**
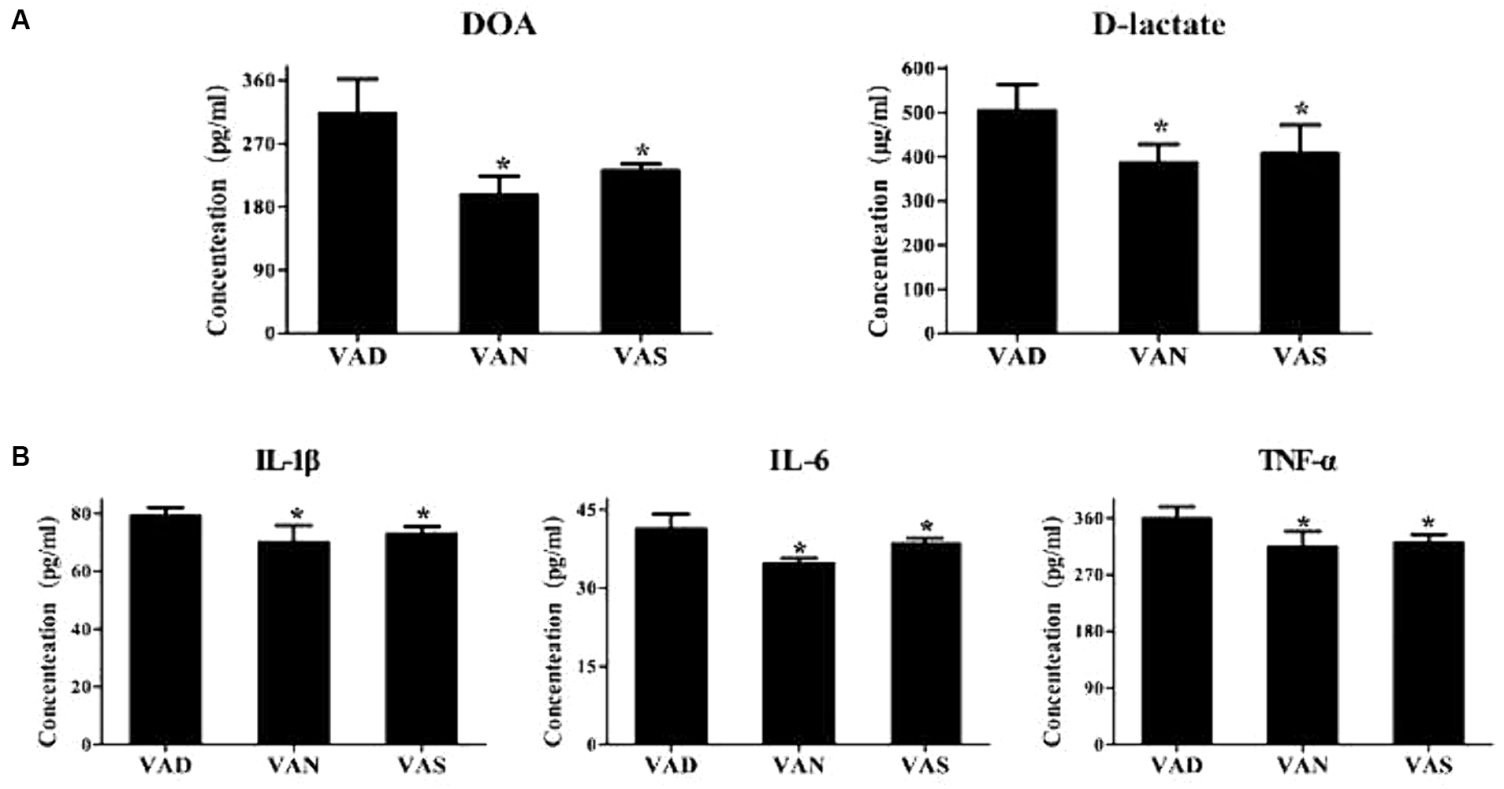
Effects of vitamin A on intestinal mucosal permeability and inflammation in APP/PS1 mice. **(A)** Comparison of diamine oxidase levels (DOA) and D-lactate, indicators of intestinal mucosal permeability, among different groups of APP/PS1 mice. All of the data were analyzed using a one-way ANOVA and they are expressed as means ± SD. *Indicates compared with the VAD group, *p* < 0.05 indicates statistical significance; **(B)** Comparison of the levels of inflammatory markers TNF-α, IL-1β, and IL-6 among different groups of APP/PS1 mice. All of the data were analyzed using a one-way ANOVA and they are expressed as means ± SD. *Indicates compared with the VAD group, *p* < 0.05 indicates statistical significance.

The levels of D-lactate and diamine oxidase, indicators of intestinal mucosal permeability, were detected in the serum of APP/PS1 mice using the ELISA method. The levels of D-lactate and diamine oxidase in the VAD group were significantly higher than those in the VAN and VAS groups, with statistical significance. Although the levels of D-lactate and diamine oxidase in the VAN and VAS groups did not show statistical significance, the VAS group had slightly higher levels of D-lactate and diamine oxidase compared to the VAN group (Figure 6A). Furthermore, to detect the levels of inflammatory cytokines TNF-α, IL-1β, and IL-6 in the serum of APP/PS1 mice using the ELISA method, it was found that the VAD group had significantly higher levels of TNF-α, IL-1β, and IL-6 compared to the VAN and VAS groups, with statistical significance. Although the levels of TNF-α, IL-1β, and IL-6 in the VAN and VAS groups did not show statistical significance, the VAS group had slightly higher levels of TNF-α, IL-1β, and IL-6 compared to the VAN group (Figure 6B).

## Discussion

Vitamin A is known to play a crucial role in maintaining neuronal plasticity and cognitive function (9). Our study evaluated a short-term dietary vitamin A could affect the development of AD-type neuropathology and consequent behavior impairment in the APP/PS1-AD mouse model. We show that the 12-week vitamin A-deficient diet resulted in decreased serum retinol levels, impaired cognition, and increased Aβ pathologies in the hippocampus of APP/PS1 mice. Conversely, a vitamin A-enriched diet led to higher serum retinol levels, preserved cognition, and reduced Aβ pathologies in the hippocampus of APP/PS1 mice. The mechanism underlying this phenomenon is attributed to the action of dietary vitamin A, which can modulate the composition and functionality of the gut microbiota. Dietary vitamin A has the ability to influence the transcriptome of the intestine, thereby affecting the regulation of intestinal permeability and the release of inflammatory factors. This impact can alter the intestinal environment, promoting or inhibiting the formation and deposition of Aβprotein, thus influencing Aβ pathology.

Dietary vitamin A affects serum retinol levels in APP/PS1 mice through the intake of provitamin A carotenoids (mainly β-carotene) or preformed vitamin A (such as retinyl esters) (10). Dietary vitamin A also affects cognitive functions and Aβ pathologies in the hippocampus of APP/PS1 mice. The findings of our study align with previous research (11–13), indicating that reduced levels of vitamin A can worsen cognitive impairment and Aβ pathology in a mouse model of Alzheimer’s disease. However, we have observed that the administration of vitamin A supplementation effectively counteracts this progression (14, 15), and surprisingly, the VAS group shows higher Aβdeposition compared to the VAN group, indicating a notable difference in Aβaccumulation. The reason for this might be related to our study following the “Chinese Dietary Reference Intakes for Residents (2013 Edition).” The Recommended Nutrient Intake (RNI) of vitamin A for adult males is 800 μg RAE per day, with a Tolerable Upper Intake Level (UL) of 3,000 μg RAE per day. By adjusting the vitamin A content from 4 IU per gram to 15 IU /g in the AIN93G diet, the level of vitamin A in the VAS group’s diet is 15 IU /g, equivalent to the UL in the human body. The long-term intake of vitamin A at the UL level can have pathological effects on the body. In this study, we assessed the levels of D-lactate and diamine oxidase, which serve as indicators of intestinal mucosal permeability. Our findings demonstrate that inadequate vitamin A levels can augment intestinal permeability, whereas vitamin A supplementation can ameliorate this phenomenon, aligning with previous research (16). This underscores the essential role of vitamin A in maintaining immune homeostasis and fostering immune tolerance within the intestinal milieu (17). Additionally, our findings indicate that a deficiency in vitamin A leads to an elevation in serum pro-inflammatory cytokines TNF-α, IL-1β, and IL-6 in APP/PS1 mice. Conversely, supplementation of vitamin A results in a decrease in these cytokines. It is worth noting that TNF-α has the ability to stimulate γ-secretase activity, which subsequently leads to an increased synthesis of Aβ peptides (18). By blocking the TNF-α pathway, the formation of Aβ plaques in the AD-animal brain can be reduced (19). Furthermore, the elevation of IL-1β has been linked to the progression and onset of AD. However, inhibiting IL-1β has shown significant potential in diminishing brain Aβ deposition in 3xTg-AD mice (20). The production and signaling of IL-6 at higher levels may be associated with cognitive decline and the formation of Aβ aggregates in AD. Additionally, this study suggests that targeting the pro-inflammatory IL-6 signaling pathway could be a potential strategy to mitigate memory deficits and metabolic disturbances in AD (21).

A dietary deficiency of vitamin A can lead to a decrease in the Shannon index. After dietary supplementation of vitamin A, the Shannon index is restored. This indicates that dietary vitamin A can impact the diversity of gut microbiota in APP/PS1 mice. This result is consistent with studies on dietary vitamin A in other AD model mice (14). The presence of certain bacteria in the gut is heightened due to the effect of dietary vitamin A, whereby specific bacterial groups exhibit either anti-inflammatory or pro-inflammatory effects. In the VAN and VAS groups, the predominant anti-inflammatory bacteria in the VAN group are Akkermansia and Verrucomicrobiales, while the anti-inflammatory bacterial communities in the VAS group belong to the genus Desulfovibrio. Conversely, the predominant pro-inflammatory bacteria in the VAD group consist of microbial communities such as Parabacteroides and Tannerellaceae. Akkermansia, a mucin-degrading bacterium, regulates host barrier function and immune response, highlighting its anti-inflammatory effects and its potential as a promising strategy for the therapy of inflammatory bowel disease by addressing reduced intestinal colonization that contributes to the development of such diseases (22). Verrucomicrobia possesses anti-inflammatory properties that contribute to intestinal health. Research has indicated a beneficial relationship between the foxp3 gene, which is responsible for promoting anti-inflammatory responses and immunity in humans (23). Desulfovibrio are sulfate-reducing bacteria have the ability to reduce sulfate to hydrogen sulfide, which, when accumulated, can lead to damage to the intestinal epithelium, causing chronic inflammation and disrupting the balance between cellular proliferation and apoptosis (24). In this enclosed niche of the intestinal system, bacteria that inhibit inflammation and bacteria that promote inflammation competitively inhibit each other’s growth, maintaining a dynamic balance in the gut. Parabacteroides, which possess physiological characteristics related to carbohydrate metabolism and the secretion of short-chain fatty acids, have recently been reported to be closely associated with host health, such as metabolic syndrome, inflammatory bowel disease, and obesity, as inflammatory mediators (25). Tannerellaceae, as oral pathogenic bacteria, have been extensively studied for their association with oral diseases, particularly chronic periodontitis. Their presence at lesion sites of periodontitis is notably high, suggesting a role in promoting inflammation (26). Additionally, in Sleep deprivation C57BL/6 J mice, the abundance of Tannerellaceae in the gut showed a positive correlation with the levels of pro-inflammatory cytokines (27).

Vitamin A and its active metabolite, RA, have been suggested to exhibit beneficial effects in Alzheimer’s disease by mechanisms that involve inhibiting the formation, elongation, and destabilizing effects of β-amyloid fibrils (28, 29). The obtained RNA sequencing results of the upper segment of the small intestine in APP/PS1 mice subjected to vitamin A intervention in various groups revealed distinct gene expression profiles associated with different vitamin A interventions in the intestinal tissue of APP/PS1 mice. The analysis of DEGs using the GO classification method demonstrated significant enrichment in various cellular processes, metabolic processes, cellular anatomical entities, intracellular activities, as well as binding and catalytic activities. This enrichment was observed when comparing the VAD group to the VAS group, the VAD group to the VAN group, and the VAS group to the VAN group. The DEGs of the pathway analysis in the small intestine of APP/PS1 mice, comparing the VAD group to the VAS and VAN groups, were subjected to KEGG and pathway enrichment analyses. These analyses revealed significant enrichment in pathways associated with carcinogenesis, drug metabolism, xenobiotic metabolism, and immune responses.

Vitamin A has been found to exert a notable influence on carcinogenesis, as supported by research indicating that mice with a deficiency in vitamin A exhibited heightened intestinal inflammation, delayed mucosal healing, and heightened immune responses. These factors collectively contributed to an increased vulnerability to colorectal cancer (CRC) (30). Additionally, it has been observed that inflammation induced by gut bacteria impacts the metabolism of all-trans retinoic acid (AtRA), resulting in diminished levels of AtRA, this reduction in AtRA levels has been implicated in the advancement of ulcerative colitis (UC) and its associated CRC (31). Moreover, the coalescence of retinol and retinol-binding protein (RBP) elicited the activation of the STRA6 oncogene, thereby substantiating its involvement in the genesis of CRC (32). In essence, the insufficiency of vitamin A expedited the progression of colitis and the development of CRC by perturbing immune responses and metabolic processes. RA, an essential metabolite of vitamin A in the intestinal tract, plays a significant role in modulating the expression and functionality of specific cytochrome P450 enzymes, thereby exerting an influence on the metabolism of select drugs. It possesses the ability to induce or inhibit the expression of these enzymes, consequently resulting in alterations in drug metabolism rates. This, in turn, can have implications for the pharmacokinetics and effectiveness of drugs, as well as the potential for drug–drug interactions (33), while also regulating the intestinal immune tolerance toward commensal bacteria and food antigens (34, 35). Notably, vitamin A deficiency has been demonstrated to impact xenobiotic-metabolizing enzymes and the body’s defense mechanisms (36).

To summarize, the aforementioned findings underscore the importance of vitamin A in the context of Alzheimer’s disease pathology and behavior within the APP/PS1 mouse model. This phenomenon is attributed to the modulation of gut microbiota by dietary vitamin A, which subsequently impacts intestinal permeability, inflammatory factors, and ultimately the formation and deposition of Aβ protein, thereby exerting an influence on Aβ pathology. These findings make a substantial contribution to the progress of understanding Alzheimer’s disease and provide valuable insights for the development of potential therapeutic strategies.

## Data availability statement

The original contributions presented in the study are publicly available.All raw RNA sequencing data have been deposited in the Dryad data repository (https://doi.org/10.5061/dryad.tmpg4f55w).

## Ethics statement

The animal studies were approved by Animal Ethics Committee of Capital Medical University (Beijing, China) (No. AEEI-2022-236). The studies were conducted in accordance with the local legislation and institutional requirements. Written informed consent was obtained from the owners for the participation of their animals in this study.

## Author contributions

Z-LW: Writing – original draft. S-JP: Methodology, Writing – review & editing. K-WZ: Data curation, Writing – review & editing. P-YL: Data curation, Writing – review & editing. P-GL: Project administration, Supervision, Writing – review & editing. CY: Funding acquisition, Project administration, Supervision, Writing – review & editing.

## Footnotes

^1^ http://www.biocloud.net
